# DNA methylation as prognostic factors in non-muscle-invasive bladder cancer: a systematic review and meta-analysis

**DOI:** 10.3389/or.2025.1679974

**Published:** 2025-11-25

**Authors:** Vishwajeet Singh, Mukul Kumar Singh, Anil Kumar, Ashutosh Shrivastava, Dinesh Kumar Sahu, Mayank Jain, Anuj Kumar Pandey

**Affiliations:** 1 Department of Urology, King George’s Medical University, Lucknow, Uttar Pradesh, India; 2 Centre for Advance Research, King George’s Medical University, Lucknow, Uttar Pradesh, India; 3 Central Research Facility, Post Graduate Institute of Child Health, Noida, Uttar Pradesh, India; 4 Department of Thoracic Surgery, King George’s Medical University, Lucknow, India; 5 Department of Biochemistry, Dr. Ram Manohar Lohia Institute of Medical Sciences, Lucknow, Uttar Pradesh, India

**Keywords:** bladder cancer, non-muscle-invasive bladder cancer, prognostic markers, DNA methylation biomarkers, predictive factor

## Abstract

**Introduction:**

Early prognostication in non-muscle-invasive bladder cancer (NMIBC) is essential for optimizing therapy and follow-up. Epigenetic mechanisms, particularly DNA methylation, have emerged as promising biomarkers for predicting disease outcome.

**Materials and Methods:**

A systematic review and meta-analysis were conducted according to Preferred Reporting Items for Systematic Reviews and Meta-Analyses (PRISMA) guidelines to evaluate the prognostic significance of promoter DNA methylation in NMIBC. Comprehensive searches of PubMed, Web of Science, Embase, MEDLINE, and the Cochrane Library (January 2010–October 2022) identified eligible studies. The Newcastle–Ottawa scale was used for quality assessment, and pooled hazard ratios with 95% confidence intervals were calculated using random-effects models.

**Results:**

Eleven studies with 3,065 NMIBC patients were analyzed. Promoter methylation was significantly associated with poor progression-free survival (pooled hazard ratios (HR) = 2.88; 95% CI = 2.03–4.09; p < 0.0001) and recurrence-free survival (pooled HR = 2.65; 95% CI = 1.93–3.63; p < 0.0001). Although overall survival showed pathway-specific variation (pooled HR = 0.96; 95% CI = 0.36–2.60; p = 0.94), methylation of adhesion and apoptosis-related genes exhibited the strongest associations. Subgroup analyses revealed a greater prognostic impact in Asian cohorts (p < 0.0001), suggesting regional differences in epigenetic susceptibility.

**Conclusion:**

Promoter DNA methylation constitutes a robust prognostic biomarker for recurrence and progression in NMIBC, with stronger effects in Asian populations. Standardization of validated gene panels, assay thresholds, and cross-regional prospective validation will be essential for clinical translation. Integrating methylation-based classifiers into risk stratification models could improve individualized management and long-term outcomes in NMIBC.

## Introduction

Bladder cancer is the 10th most prevalent cancer, and its cases are continuously on the rise in developed and developing countries. It ranked fourth among men and 12th among women and accounts for 0.2 million deaths worldwide (1). Non-muscle-invasive bladder cancer (NMIBC) is a confined form and one of the clinically significant subtypes of bladder cancer (2). After the standard treatment with transurethral resection of the bladder tumor (TURBT) followed by intravesical *Bacillus* Calmette–Guérin (BCG) immunotherapy, recurrence occurs in up to 60%–70% of patients, and 10%–30% disease progression to MIBC (3–5). BCG-non-responsive NMIBC presents a challenging scenario, as treatment failure necessitates radical cystectomy, a morbid procedure with impacts on quality of life (6, 7). Despite the availability of clinicopathological risk stratification tools, such as tumor grade, stage, and multiplicity, these parameters remain insufficient to predict recurrence, progression, or overall survival (OS) (8, 9). Hence, identifying reliable molecular biomarkers that can improve prognostic precision and targeted therapy in NMIBC patients is an urgent need.

Among molecular mechanisms implicated in bladder carcinogenesis, epigenetic regulation has emerged as a crucial driver of tumor development and progression (10–12). DNA methylation is extensively studied and involves the addition of a methyl group to cytosine residues within CpG islands in gene promoters, leading to transcriptional silencing (13). In bladder cancer, tumor-suppressor genes such as *RASSF1A*, *RUNX3*, *CDH13*, and *HOXA9* have been reported to undergo promoter hypermethylation, resulting in loss of tumor-inhibitory function (14, 15). Such methylation events contribute to cell cycle dysregulation, inhibition of apoptosis, and enhanced tumor invasiveness (16, 17). Despite multiple studies exploring individual methylation biomarkers in NMIBC, the findings remain inconsistent due to variations in sample size, patient characteristics, methodologies, and analytical approaches (18). This inconsistency highlights the need for a systematic synthesis of available evidence to clarify the prognostic utility of DNA methylation in NMIBC. Therefore, the present systematic review and meta-analysis were undertaken to comprehensively evaluate the existing literature and determine the overall prognostic significance of DNA methylation in NMIBC by quantitatively analyzing its association with key clinical outcomes, recurrence-free survival (RFS), progression-free survival (PFS), and overall survival (OS). This study aims to establish whether DNA methylation can serve as a reliable biomarker for patient stratification and clinical decision-making in the management of non-muscle-invasive bladder cancer.

## Methods

### Search strategy

We followed the recommendations established by the Preferred Reporting Items for Systematic Reviews and Meta-Analyses (PRISMA) group in 2020. The International Prospective Register of Systematic Reviews has documented its registration (ID: CRD42022376063). The selected eligible articles, published between January 2010 and October 2022, were included after a comprehensive search of PubMed, Web of Science, Embase, and the Cochrane Library databases.

Various keywords and Medical Subject Headings (MeSH) terms, including bladder cancer, urothelial cancer, carcinoma *in situ*, non-muscle-invasive bladder cancer, low-grade bladder cancer, transitional cell carcinoma, low-risk bladder cancer, methylation, and DNA methylation, were used to search the relevant literature.

### Selection and eligibility criteria of the studies

The studies were selected based on following criteria: (i) articles that were published in English in scholarly journals or periodical literature; (ii) studies whose histologic type of tumor was bladder carcinoma or NMIBC; (iii) studies that investigated the association between DNA methylation or biomarkers and survival outcomes (overall survival and recurrence-free survival) of the bladder cancer patients; (iv) articles that reported the sample size, hazard ratios, and 95% CIs. However, articles were excluded from this meta-analysis if any of the following criteria were met: i) they were primary research or studies conducted on animals; (ii) meta-analyses, systematic reviews, case reports, conference proceedings, unpublished, or ongoing research; (iii) data were unavailable or insufficient; (iv) the articles were written in a language other than English; and (v) the articles were irrelevant or overlapped with other research articles. Following the elimination of duplicates, two separate reviewers, AK and MKS, evaluated the eligibility of the studies and filtered them through their abstracts and titles. This helped us achieve a higher level of dependability. Following this, the complete texts of the articles were evaluated to see whether they met the inclusion criteria and were validated for their relevance. All of the reviewers came together and reached a decision by consensus if a dispute arose.

### Data extraction and quality assessment

Two reviewers, AK and MKS, independently extracted and assessed study data, while a third reviewer, VS, resolved inconsistencies. Each study was assessed for clear goals, randomization methods, blinding of interventions, and participants, determining who was eligible for the intervention, giving enough information about the intervention so that it could be repeated, effect size, details of long-term follow-up and changes that lasted, analysis of confounding variables, power analysis, the definition of all outcomes, reliable measurement tools, results that could be used to measure, and appropriate statistical analysis. First author’s last name, publication year, area, study design, sample size and age, DNA methylation/biomarkers, follow-up length, association cutoff, and survival outcomes were tallied. The Newcastle–Ottawa scale was used to measure study quality (NOS). The scale has three parts: selection (0–4 points), comparability (0–2 points), and result (0–4 points) (awarded 0–3 points). The maximum NOS score was 9; ≥6 was high-quality research.

### Statistical analysis

Review Manager (RevMan) version 5.4 (RevMan 5; The Nordic Cochrane Center, The Cochrane Collaboration, Copenhagen, Denmark) was used to conduct a meta-analysis of prognostic indices such OS, RFS, and PFS The link between DNA methylation and bladder cancer outcomes was determined by pooling the hazard ratios (HRs) from the included studies, together with their associated 95% CIs. The heterogeneity of the included articles was analyzed using the Cochran’s Q test and the Higgins I^2^ statistic. In cases where considerable heterogeneity was indicated by the Q test (p = 0.10) or the I^2^-test (I2 > 50%), a random-effect model (the DerSimonian–Laird technique) was used. Other than that, the Mantel–Haenszel approach (fixed-effect model) was explored.

## Results

### Study selection

**FIGURE 1:**
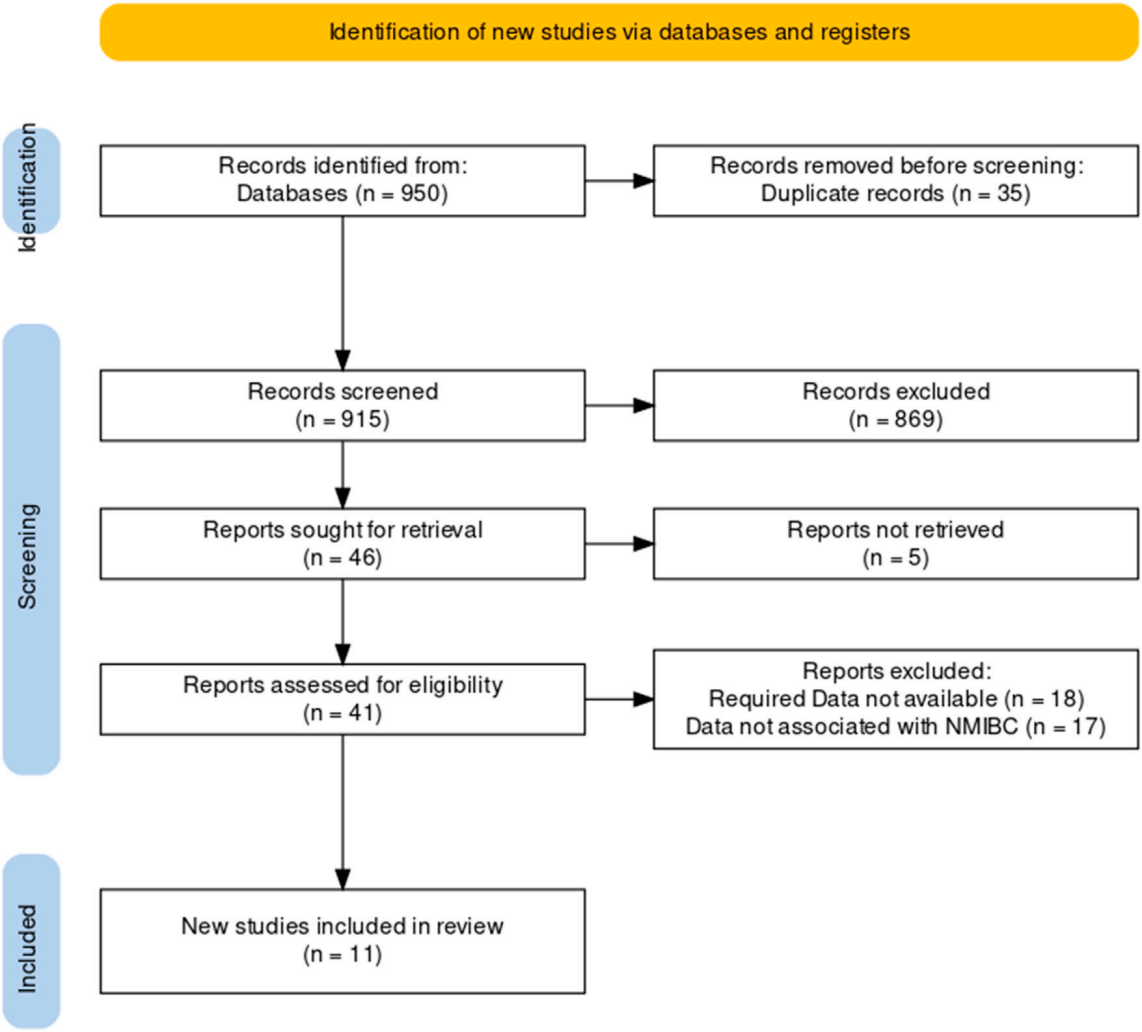
Preferred Reporting Items for Systematic Reviews and Meta-Analyses (PRISMA) flow diagram for the study selection process.

Our article search found a total of 950 articles, including duplicates and articles with incomplete data. The independent reviewer screened the articles based on their titles and abstracts, excluding 869 articles, and 5 articles were not retrieved. The full text review of the screened articles was completed, and 46 articles were assessed. As shown in Figure 1, 11 studies were considered eligible for inclusion in the systematic review and meta-analysis (19).

### Study characteristics

The meta-analysis includes 11 eligible articles for NMIBC with a sample size of 3,065 patients. The sample sizes range from 55 to 1,249 participants, and the participants’ ages range from 10 to 96, with follow-up periods of 3 months to 190 months. The eligible studies were considered from 2010 to 2022 (12 years) with geographic diversification and type of study focusing on NMIBC (Table 1). The prognostic outcome of the study included ten articles for PFS (20–29), nine articles on RFS (20, 21, 23–28, 30), and two articles on OS of NMIBC (28, 30). The included studies discussed various DNA methylation biomarkers for the prognosis of bladder cancer and treatment strategy.

**TABLE 1:** Characteristics of included studies.

Reference	Country/Study duration	Study design	Sample size	Median age (years)	Treatment	DNA methylation biomarker(s)	Analysis method	HR (95% CI)	Follow-up (months)	Key findings/Conclusion
(20)	Spain (1989–2008)	Prospective cohort	170 T1G3 NMIBC (108 BCG-treated, 72 recurrence, 36 progression, and 24 deaths)	71.5 (28–94)	TURBT; BCG (Connaught strain) or MMC 40 mg/50 mL	*Myopodin*	Univariate and multivariate	PFS HR = 11.23 (1.53–82.36), p = 0.017; RFS HR = 2.54 (1.21–5.35), p = 0.01	52.5 (3–190)	*Myopodin* methylation is associated with recurrence, progression, and shorter disease-free survival (DFS) in T1G3 NMIBC treated with BCG
(21)	South Korea (N/R)	Prospective	179 NMIBC (69 recurrence and 18 progression)	67 (24–91)	First TURBT; second TURBT in 49 cases; intravesical (75 BCG and 34 MMC)	*RUNX3* and *MGC17624*	Univariate and multivariate	PFS HR = 20.08 (1.72–234.18), p = 0.017; RFS HR = 54.23 (4.04–727.40), p = 0.003	57.8 (9.1–189.7)	Combined *RUNX3* and *MGC17624* methylation predicts NMIBC progression at diagnosis
(24)	South Korea (1995–2009)	Prospective cohort	301 (186 NMIBC and 115 MIBC)	67 (10–91)	TURBT; 76 BCG; 29 MMC; 115 radical cystectomy; 54 cisplatin	*RASSF1A*	Multivariate	PFS HR = 8.56 (1.55–47.36), p = 0.014; RFS HR = 6.14 (1.04–36.41), p = 0.046	51.4 (mean)	*RASSF1A* methylation is a potential prognostic marker for recurrent NMIBC
(23)	South Korea (1995–2010)	Prospective case–control	187 (181 NMIBC and 6 controls)	Case = 56.3 ± 25.5; control = 64.3 ± 13.8	TURBT	*HOXA9*, *ISL1*, *ALDH1A3*, and *EOMES*	Multivariate and univariate	PFS HRs: *ISL1* = 3.30 (1.05–12.92), p = 0.041; *ALDH1A3* = 3.55 (1.07–14.22), p = 0.039; RFS HRs: *HOXA9* = 1.87 (1.14–3.47), p = 0.032; *ISL1* = 1.71 (1.05–3.47), p = 0.039; *ALDH1A3* = 1.68 (1.02–3.16), p = 0.044	35.8 (6.1–183.3)	*HOXA9*, *ISL1*, and *ALDH1A3* methylation serves as independent prognostic indicators for NMIBC recurrence and progression
(28)	China (2004–2008)	Prospective case–control	233 NMIBC and 43 controls (bladder stone patients)	<65/≥65	TURBT	*PCDH8*	Multivariate	OS HR = 3.02 (1.54–5.90), p = 0.001; PFS HR = 2.52 (1.65–7.43), p = 0.004; RFS HR = 4.74 (1.87–12.05), p < 0.0001	N/R	*PCDH8* methylation is common in NMIBC; it is an independent predictor of recurrence, progression, and OS
(27)	China (2004–2007)	Prospective cohort	178 TCC NMIBC	<65/≥65 (p = 0.077)	TURBT + 1 cycle MMC	*CDH13*	Multivariate	PFS HR = 6.56 (2.24–21.71), p = 0.0016; RFS HR = 5.15 (2.07–20.18), p = 0.0043	N/R	*CDH13* methylation is a frequent and independent predictor for NMIBC recurrence and progression
(25)	Republic of Korea (1995–2010)	Prospective case–control	136 (128 NMIBC and 8 controls)	NC = 59 ± 22.2; NMIBC = 64 ± 13.4	TURBT	*PRAC*	Multivariate	PFS HR = 9.53 (1.17–77.50), p = 0.035; RFS HR = 2.65 (1.24–5.67), p = 0.012	N/R	*PRAC* methylation is significantly associated with the high grade/stage; it is an independent predictor of recurrence and progression
(22)	South Korea (N/R)	Case–control	55 NMIBC + adjacent normal tissue	64 (24–84)	TURBT, BCG, and MMC	*RUNX3*	Multivariate	PFS HR = 5.69 (1.06–30.56), p = 0.043	76 (14–164)	*RUNX3* methylation in adjacent urothelium predicts progression in NMIBC
(26)	Republic of Korea (1995–2010)	Prospective case–control	136 (128 NMIBC and 8 controls)	NC = 59 ± 22.2; BC = 62.6 ± 14.5	TURBT ± 5-Aza-CdR (*in vitro*)	*RSPH9*	Multivariate	PFS HR = 8.25 (1.26–54.09), p = 0.028; RFS HR = 3.02 (1.61–5.67), p = 0.001	N/R	*RSPH9* methylation is an independent indicator of NMIBC recurrence and progression
(29)	Multicentric (Europe: Denmark, Germany, Serbia, Spain, Sweden, and Netherlands; 1979–1989)	Prospective	1,239 NMIBC (276 low risk, 273 intermediate, and 555 high risk)	Mean = 70 (21–96)	TURBT ± BCG	*GATA2*, *TBX2*, *TBX3*, *ZIC4* (+ *FGFR3*, *TERT*, *PIK3CA*, or *RAS* mutation)	Univariate and multivariate	PFS HR: *GATA2*: 2.04 (1.01–4.10), p = 0.046; *TBX2*: 1.36 (0.65–2.82), p = 0.41; *TBX3*: 1.71 (0.86–3.43), p = 0.13; *ZIC4*: 1.43 (0.70–2.89), p = 0.33	Median: 27 (0–81)	*GATA2* and *TBX3* methylation with wild-type *FGFR3* significantly predicts progression
(30)	Spain (1989–2009)	Retrospective–prospective cohort	251 (pTa LG = 79, pT1 LG = 81, and pT1 HG = 91)	<65/≥65	TURBT ± BCG (Connaught strain)	*PAX5A*, *RB1*, *WT1*, *BRCA1*, *PYCARD*, and *GSTP1*	Univariate and multivariate	OS HRs: *GSTP1* = 0.59 (0.037–0.95), p = 0.028; *PYCARD* = 0.54 (0.34–0.85), p = 0.008; *RFS* HRs: *PAX5A* = 1.61 (1.11–2.33), p = 0.01; *RB1* = 2.31 (1.23–4.33), p = 0.008	Up to 24 months	*PAX5A* and *RB1* methylation predicts recurrence; *GSTP1* and *PYCARD* associate with a favorable outcome

### DNA methylation biomarkers and progression-free survival (PFS) with NMIBC

**FIGURE 2:**
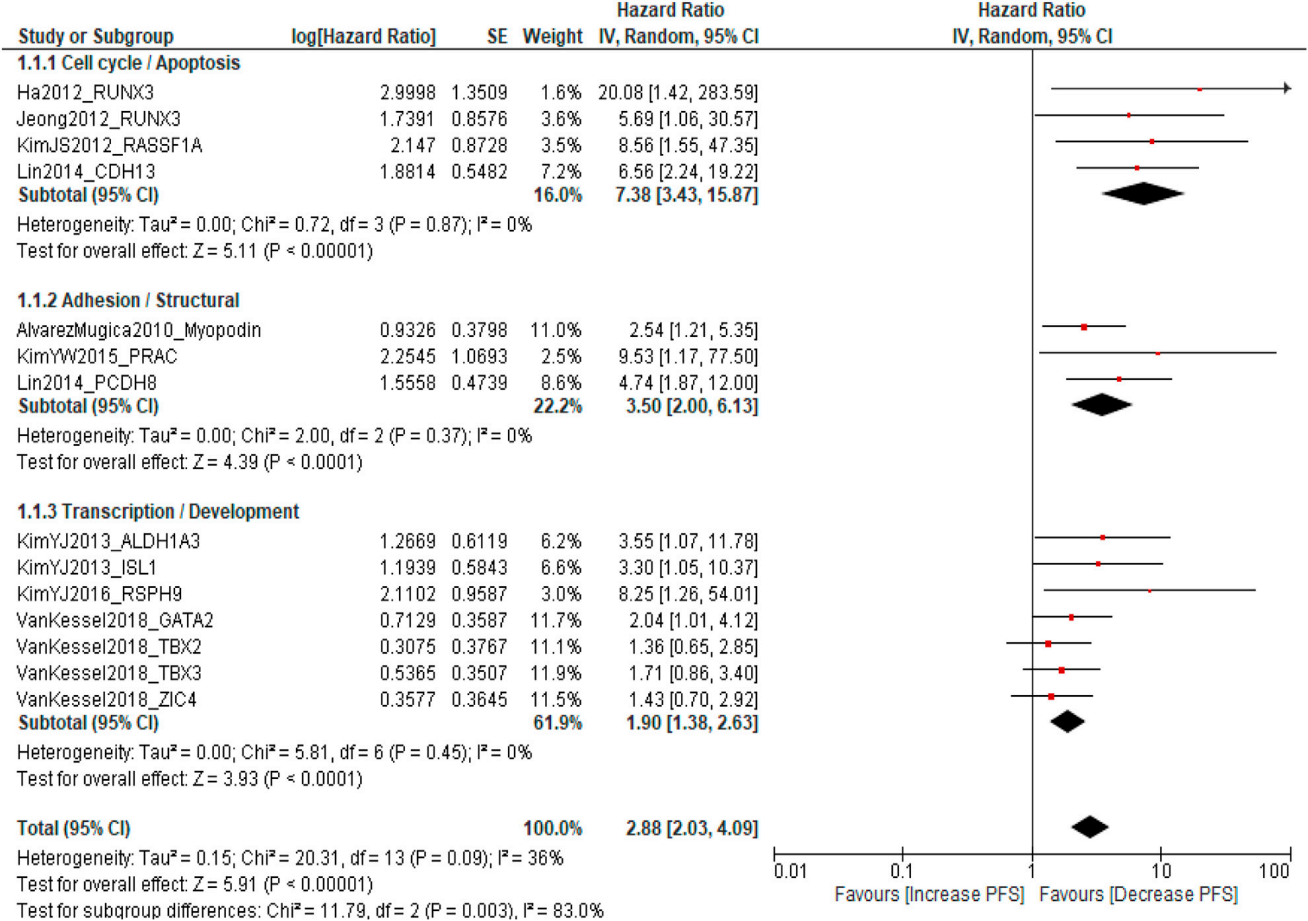
Forest plot for DNA methylation biomarkers and progression-free survival.

A total of ten published studies comprising 2,814 patients with NMIBC were included in the analysis evaluating the association between promoter DNA methylation and PFS. The pooled analysis demonstrated that DNA methylation was significantly associated with poorer PFS (pooled HR = 2.88; 95% CI = 2.03–4.09; p < 0.0001), indicating that patients with methylated genes had a threefold higher risk of disease progression compared with those without methylation. Overall heterogeneity was moderate (I^2^ = 36%, p = 0.09), suggesting acceptable inter-study variability. Subgroup analysis according to molecular pathways revealed that cell cycle-/apoptosis-related genes (*RUNX3*, *RASSF1A*, and *CDH13*) showed the strongest association with progression (pooled HR = 7.38; 95% CI = 3.43–15.87; I^2^ = 0%), followed by adhesion/structural genes (*Myopodin*, *PRAC*, and *PCDH8*) (pooled HR = 3.50; 95% CI = 2.00–6.13; I^2^ = 0%) and transcription/developmental genes (*ALDH1A3*, *ISL1*, *RSPH9*, *GATA2*, *TBX2*, *TBX3*, and *ZIC4*) (pooled HR = 1.90; 95% CI = 1.38–2.63; I^2^ = 0%). A significant difference was observed among subgroups (p = 0.003), indicating pathway-specific variability in methylation effects, while sensitivity analysis confirmed the robustness of the pooled estimates Supplementary Table S1. These findings indicate that promoter DNA methylation, particularly involving genes regulating cell cycle control, apoptosis, and cell adhesion, is consistently associated with an increased risk of progression and reduced PFS in NMIBC patients (Figure 2).

### Sensitivity analysis of PFS

A leave-one-out sensitivity analysis was conducted to evaluate the robustness of the pooled PFS estimate. Sequential exclusion of each study from the meta-analysis yielded pooled hazard ratios ranging from 2.64 to 3.00, with moderate and stable heterogeneity (I^2^ = 30–44%) and consistently significant associations (all p < 0.15). Exclusive of Kim et al. (2013), ALDH1A3 and ISL1 slightly increased heterogeneity (I^2^ = 44%, p = 0.05) but did not materially alter the overall effect size.

These findings confirm that no individual study disproportionately influenced the pooled estimate, indicating the stability and reliability of the meta-analytic results (Supplementary Table S1).

### Association between DNA methylation and recurrence-free survival (RFS)

**FIGURE 3:**
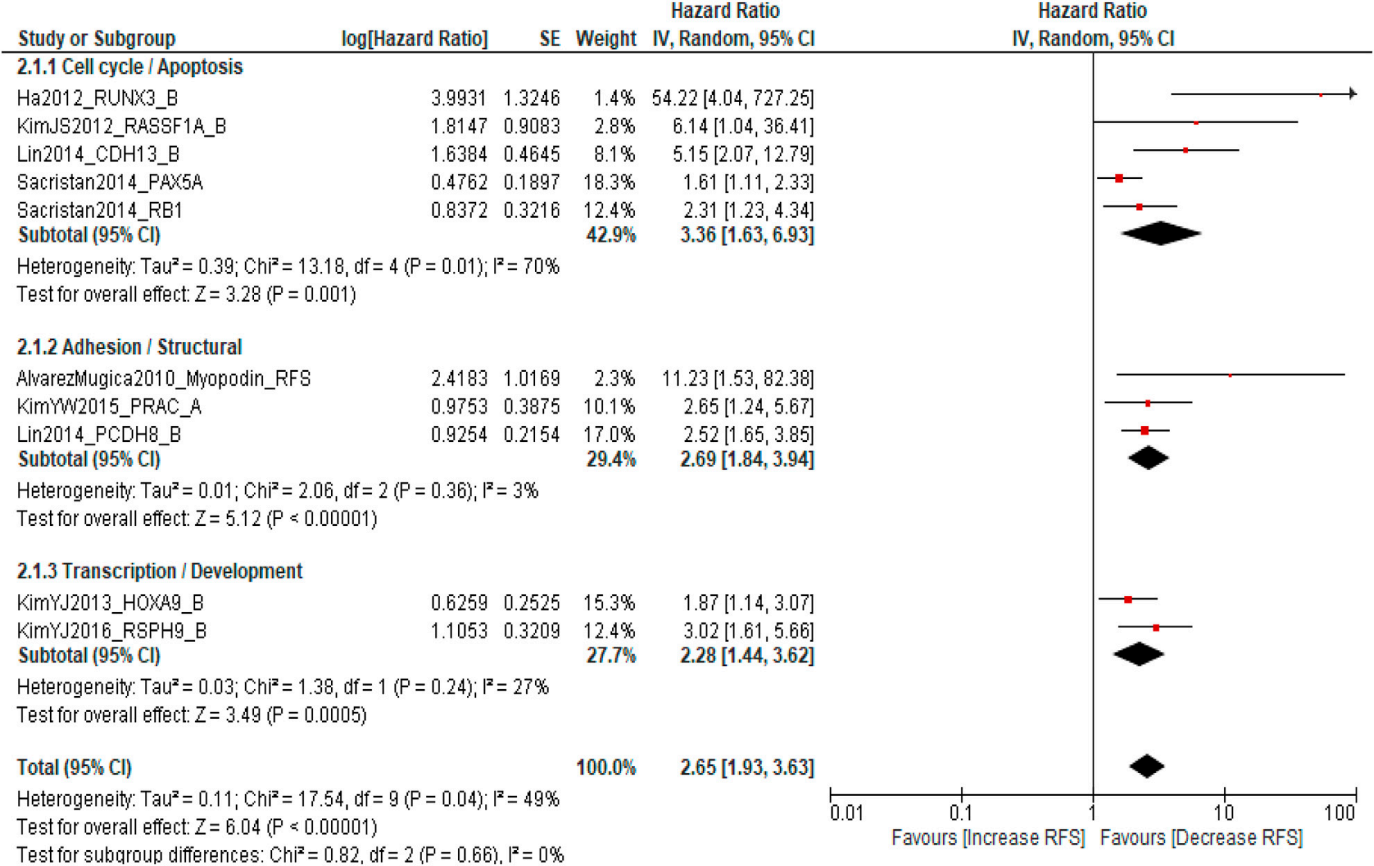
Forest plot showing the pooled hazard ratios for RFS according to molecular pathway subgroups.

A total of 1,771 patients with NMIBC were included in the meta-analysis evaluating the association between DNA methylation biomarkers and RFS. The pooled analysis demonstrated that DNA methylation was significantly associated with an increased risk of recurrence (pooled HR = 2.65; 95% CI = 1.93–3.63; p < 0.00001), with moderate heterogeneity (I^2^ = 49%, p = 0.04). Subgroup analysis according to molecular pathways revealed that cell cycle-/apoptosis-related genes (*RUNX3*, *RASSF1A*, *CDH13*, *PAX5A*, and *RB1*) showed the strongest association with recurrence (pooled HR = 3.36; 95% CI = 1.63–6.93; I^2^ = 70%), followed by adhesion/structural genes (*Myopodin*, *PRAC*, and *PCDH8*) (pooled HR = 2.69; 95% CI = 1.84–3.94; I^2^ = 3%) and transcription/developmental genes (*HOXA9* and *RSPH9*) (pooled HR = 2.28; 95% CI = 1.44–3.62; I^2^ = 27%). A non-significant difference was observed among subgroups (p = 0.66). The findings show promoter DNA methylation of genes regulating cell cycle, apoptosis, and cell adhesion is associated with an increased recurrence risk in NMIBC (Figure 3).

### Sensitivity analysis for recurrence-free survival

A leave-one-out sensitivity analysis was performed to assess the robustness of the pooled estimate for RFS. Sequential exclusion of each study produced pooled hazard ratios ranging from 2.43 to 3.10, with moderate and consistent heterogeneity (I^2^ = 32–54%) and sustained statistical significance (p ≤ 0.10). Exclusion of Ha et al. (2012), RUNX3 slightly reduced the pooled hazard ratio (HR = 2.43; 95% CI = 1.87–3.16) and heterogeneity (I^2^ = 32%), suggesting a minor influence of this study on inter-study variability. However, the overall direction and magnitude of association remained unchanged. These findings confirm that no individual study disproportionately influenced the meta-analytic outcome, indicating the stability and reliability of the association between promoter DNA methylation and shorter RFS in NMIBC (Supplementary Table S2).

### Overall survival (OS)

**FIGURE 4:**
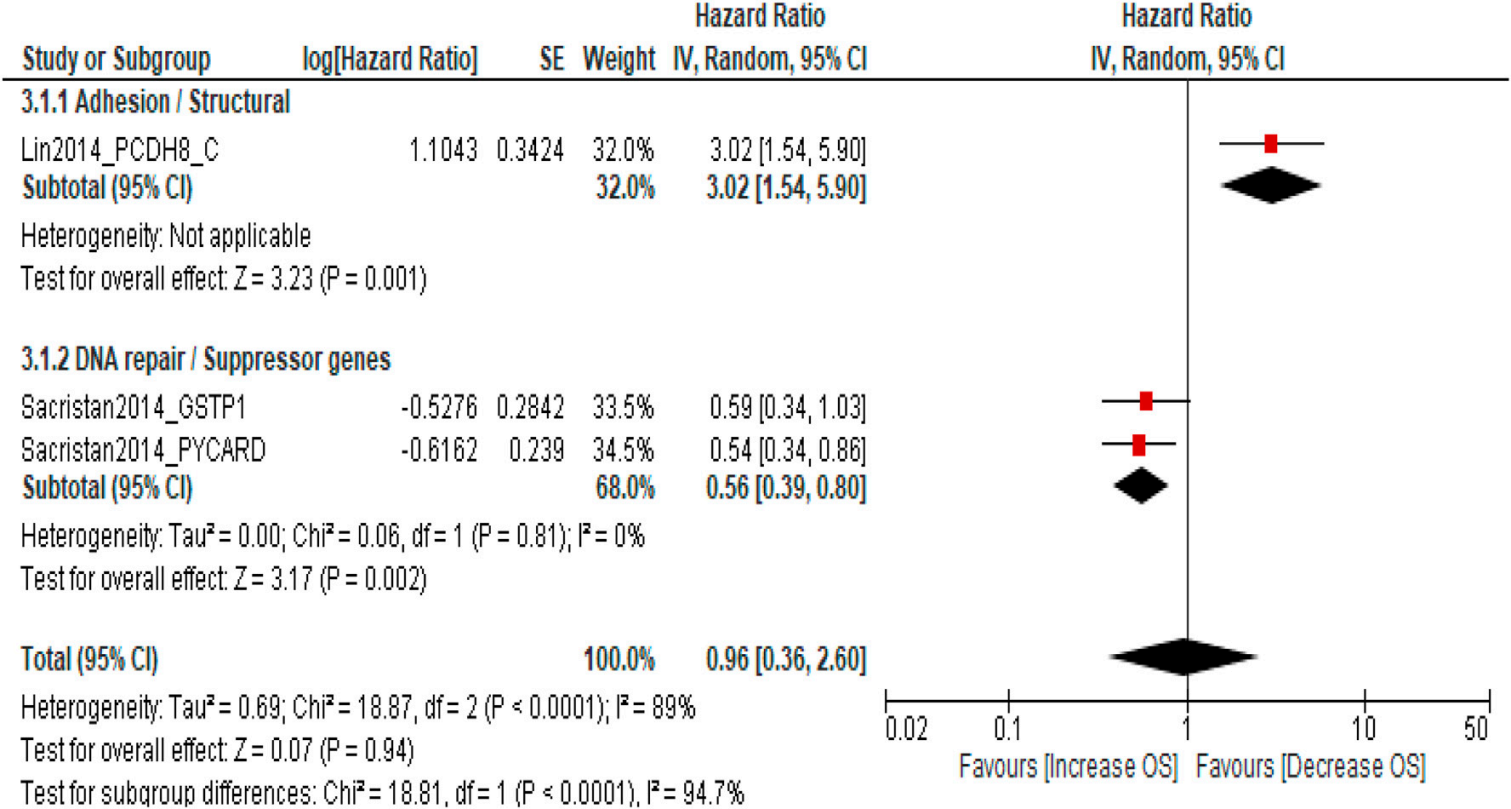
Forest plot showing pooled hazard ratios (HRs) for overall survival (OS) according to molecular pathway subgroups.

Three studies involving 484 patients with NMIBC evaluated the association between DNA methylation biomarkers and OS. The pooled meta-analysis showed no significant association between promoter methylation and OS (pooled HR = 0.96; 95% CI = 0.36–2.60; p = 0.94), with high heterogeneity across studies (I^2^ = 89%). Subgroup analysis by molecular pathway revealed contrasting trends: adhesion/structural genes (*PCDH8*) were associated with poorer OS (HR = 3.02; 95% CI = 1.54–5.90; p = 0.001), whereas DNA repair/suppressor genes (*GSTP1* and *PYCARD*) were linked to a favorable prognosis (pooled HR = 0.56; 95% CI = 0.39–0.80; p = 0.002). However, the difference between subgroups was statistically significant (p < 0.0001), indicating marked pathway-specific heterogeneity. The results suggest individual gene pathways may influence OS differently; the overall pooled estimate does not demonstrate a consistent survival impact of DNA methylation in NMIBC (Figure 4). Sensitivity analysis was not performed as only three studies were available, and the pooled estimate was not statistically significant. The limited number of studies and high heterogeneity (I^2^ = 89%) preclude a reliable robustness assessment for this outcome.

### Subgroup analysis by geographical region

To evaluate geographical variability in the prognostic role of DNA methylation, subgroup analyses were performed for PFS, RFS, and OS. The pooled HR for PFS was significantly higher in Asian cohorts (HR = 5.36; 95% CI = 3.40–8.45; p < 0.00001) than in European cohorts (HR = 1.78; 95% CI = 1.30–2.44; p = 0.0003), indicating a stronger prognostic effect of promoter methylation in Asian populations (p < 0.0001, I^2^ = 36%). A similar pattern was observed for RFS, where Asian studies (HR = 2.68; 95% CI = 2.05–3.50; p < 0.00001) demonstrated a greater effect than European studies (HR = 1.85; 95% CI = 1.35–2.54; p = 0.0001), with moderate heterogeneity (I^2^ = 54%) but consistent direction of association across regions.

In contrast, OS showed divergent results, with European cohorts suggesting a protective association for unmethylated promoters (HR = 0.56; 95% CI = 0.39–0.80; p = 0.002) and the single Asian study indicating higher mortality with methylation (HR = 3.02; 95% CI = 1.54–5.90; p = 0.001). These findings highlight region-specific differences potentially driven by genetic, environmental, and methodological factors, underscoring the need for globally representative and standardized validation of methylation biomarkers in NMIBC prognostication (Supplementary Figures S1–S3).

### Publication bias

**FIGURE 5:**
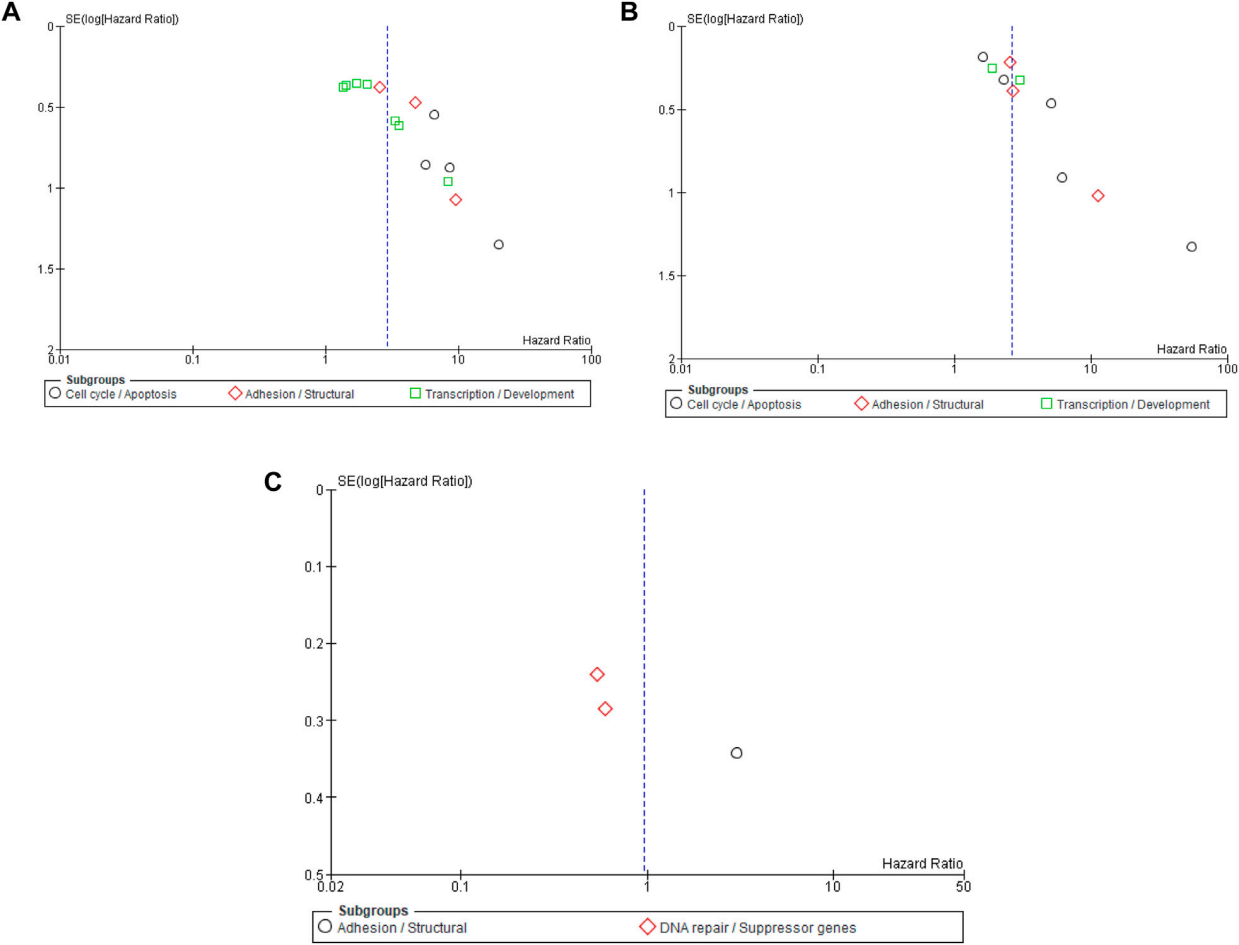
**(A)** Funnel plot for the risk bias in DNA methylation biomarkers and progression-free survival (PFS). **(B)** Funnel plot for the risk bias in DNA methylation biomarker and recurrence-free survival (RFS). **(C)** Funnel plot for the risk bias in DNA methylation biomarkers and overall survival (OS).

Publication bias was evaluated using funnel plot symmetry and Egger’s regression analysis for PFS, RFS, and OS (Figures 5A–C). Funnel plots for PFS and RFS displayed mild asymmetry, suggesting potential small-study effects, whereas the OS plot showed marked asymmetry, likely due to the limited number of included studies. Egger’s regression confirmed evidence of small-study effects for PFS (intercept = 2.73, 95% CI 1.64–3.82, p = 0.00015) and RFS (intercept = 2.48, 95% CI 1.41–3.56, p = 0.00070), indicating possible publication bias favoring studies with significant findings. Egger’s testing was not performed for OS because the small number of studies (n < 10) renders such analyses unreliable. In addition to regional influences, publication bias was assessed using geographical subgroup funnel plots for PFS, RFS, and OS. For PFS (Supplementary Figure S4), mild right-sided asymmetry was noted, with Asian studies showing higher hazard ratios and smaller standard errors, while European studies clustered symmetrically around the central axis, suggesting greater homogeneity. For RFS (Supplementary Figure S5), the distribution appeared largely symmetrical across both regions, indicating minimal small-study effects. In contrast, the OS funnel plot (Supplementary Figure S6) demonstrated notable asymmetry, mainly reflecting the limited number of studies rather than true regional bias.

### Quality assessment

The Newcastle–Ottawa scale (NOS) was used for the quality assessment of the included studies; see Table 2. The included studies scored 7–9, which implies the quality of the eligible studies was high (31).

**TABLE 2:** Quality assessment of the included study assessed by the Newcastle–Ottawa scale.

Study		Selection		Comparability		Outcome			Total
	Representativeness of the exposed cohort	Selection of the non-exposed cohort	Ascertainment of exposure	Demonstrates that the outcome of interest was not present at the start of the study	Comparability of cohorts based on the design or analysis	Assessment of outcome	Was the follow-up long enough for outcomes to occur?	Adequacy of follow-up of cohorts	
Full score	**1**	**1**	**1**	**1**	**2**	**1**	**1**	**1**	**9**
Alvarez-Múgica, 2010	1	1	1	1	2	1	1	1	9
Yun-Sok Ha, 2011	1	1	1	1	2	1	1	1	9
Ji Sang Kim, 2012	1	1	1	1	2	1	1	1	9
Kim YW, 2015	1	1	1	1	2	0	1	1	8
Yong-June Kim, 2013	1	1	1	1	2	1	0	1	8
Young-Won Kim, 2015	1	1	1	1	2	1	1	1	9
Ying-Li Lin, 2014	1	1	1	1	2	1	1	1	9
Ying-Li Lin, Pei-Gen Xie, 2014	1	1	1	1	2	1	1	1	9
Raquel Sacristan, 2014	1	1	1	1	2	1	1	1	9
Kim E.M. van Kessel, 2018	1	1	1	1	2	1	1	1	9
Jeong P. min 2012	1	1	1	1	2	1	1	1	9

Full score is the criteria for the maximum marks can be given under each subsections as mentioned are marked in bold.

## Discussion

This comprehensive meta-analysis, encompassing 3,065 patients from 11 studies, systematically evaluated the prognostic role of DNA methylation biomarkers in NMIBC. The findings provide robust evidence that promoter DNA methylation is significantly associated with adverse clinical outcomes, including reduced PFS and RFS, while its association with OS appears pathway-specific. These results underscore the crucial role of epigenetic dysregulation in driving NMIBC heterogeneity and highlight the clinical potential of methylation-based biomarkers for personalized risk stratification. The pooled hazard ratio for PFS (HR = 2.88; 95% CI = 2.03–4.09; p < 0.0001) and RFS (HR = 2.65; 95% CI = 1.93–3.63; p < 0.00001) demonstrates a consistent, nearly threefold increase in risk of disease progression and recurrence among patients with promoter-hypermethylated genes. These associations were particularly strong for cell cycle-/apoptosis-related genes (*RUNX3*, *RASSF1A*, and *CDH13*), suggesting that epigenetic silencing of key regulatory checkpoints facilitates early tumor recurrence and progression. RUNX3 methylation leads to impaired TGF-β signaling and cell cycle arrest, while RASSF1A inactivation disrupts Ras/Raf-mediated apoptosis and microtubule stability (32). Similarly, CDH13, a calcium-dependent adhesion molecule, maintains epithelial integrity, and its methylation enhances invasion and epithelial–mesenchymal transition (33). The uniform directionality across studies and subgroups (I^2^ = 0%) reinforces the biological validity of these associations. These data establish that DNA methylation serves as an independent, reproducible determinant of disease progression in NMIBC (20, 24, 27). Genes linked to adhesion and structural regulation (*Myopodin*, *PRAC*, and *PCDH8*) were also significantly associated with reduced PFS and RFS, indicating that epigenetic disruption of cell–cell adhesion and cytoskeletal architecture contributes to early tumor dissemination (34). Myopodin methylation, frequently observed in recurrent tumors, correlates with impaired actin organization and metastatic potential. Similarly, PCDH8 acts as a cell adhesion molecule and tumor suppressor, and its hypermethylation promotes detachment, invasion, and reduced survival (35). The identification of these adhesion-associated genes highlights the multifaceted role of methylation in NMIBC biology, encompassing not only proliferative signaling but also spatial and structural deregulation.

Although the pooled estimate for OS was not statistically significant (HR = 0.96; 95% CI = 0.36–2.60; p = 0.94), the subgroup analysis revealed marked pathway-specific divergence. Methylation of adhesion/structural genes such as *PCDH8* correlated with poorer OS (HR = 3.02; 95% CI = 1.54–5.90; p = 0.001), consistent with their established tumor-suppressive role. Conversely, methylation of DNA repair and suppressor genes (*GSTP1* and *PYCARD*) appeared protective (HR = 0.56; 95% CI = 0.39–0.80; p = 0.002), a paradoxical finding that may reflect compensatory methylation of stress-response genes or differing treatment sensitivities. The significant heterogeneity between pathways (p < 0.0001; I^2^ = 89%) underscores that the prognostic relevance of methylation is gene- and context-dependent rather than uniformly deleterious. These nuanced results echo recent genomic and epigenomic classifications of NMIBC that emphasize pathway-specific molecular evolution rather than a single linear progression model. The interplay between methylation and known oncogenic mutations such as *FGFR3*, *TERT*, and *HRAS* further refines our understanding of NMIBC pathogenesis. Activating *FGFR3* mutations, present in up to 70% of low-grade NMIBC, are frequently accompanied by CpG methylation of cell cycle regulators, compounding proliferative signaling (36). Van Kessel et al. demonstrated that combined FGFR3 mutation and GATA2/TBX3 methylation defines a low-grade molecular subset with distinct recurrence trajectories (29). Similarly, TERT promoter mutations synergize with hypermethylation of telomere-maintenance genes (*hTERT*, *PCDH17*, *TWIST1*, and *OTX1*) to enhance immortalization and genomic instability (37, 38). The present meta-analysis reinforces that methylation signatures should be interpreted in conjunction with driver mutations to achieve optimal prognostic accuracy.

The immunological dimension of methylation-mediated tumor behavior is another critical consideration. Epigenetic silencing of antigen-presentation and interferon-responsive genes promotes immune evasion, potentially influencing BCG responsiveness. The CD3^+^ and CD8^+^ tumor-infiltrating lymphocytes (TILs) were independently associated with reduced recurrence risk (OR = 5.8 and OR = 3.9, respectively) (39, 40). The density of CD103^+^CD8^+^ cells in the tumor–stroma interface correlated strongly with improved RFS, suggesting that methylation status shapes the immune landscape and immunotherapeutic response (41). Integrating immune markers with methylation profiling may therefore enhance risk prediction and therapeutic tailoring, particularly in the era of checkpoint inhibitors and intravesical immunotherapy (42). The emergence of liquid-biopsy methylation assays represents a transformative advance in NMIBC monitoring. Urinary methylation panels such as Bladder EpiCheck™ and AssureMDx have achieved sensitivity exceeding 80% and AUC values approaching 0.9 for recurrence detection (43, 44). More recently, assays targeting methylated GHSR, MAL, SOX1-OT, and HIST1H4F loci have demonstrated comparable diagnostic accuracy in multi-institutional validation cohorts (45). These non-invasive platforms enable real-time surveillance and complement cystoscopy, potentially reducing procedural burden while maintaining diagnostic fidelity. The strong correlation between tissue and urinary methylation profiles supports the translational relevance of our findings, suggesting that promoter hypermethylation markers could soon be integrated into clinical follow-up algorithms. Although regional subgroup analysis revealed stronger prognostic effects of promoter methylation in Asian NMIBC cohorts (HR = 5.36) than in European cohorts (HR = 1.78), this likely reflects genetic, epigenetic, and environmental differences influencing methylation susceptibility and tumor biology. These findings highlight the need for multi-ethnic validation using standardized assays for global applicability (46, 47).

The biological rationale linking methylation and clinical outcome is increasingly supported by integrated multi-omics approaches. Whole-genome bisulfite sequencing and methylome clustering have delineated reproducible epigenetic subtypes of NMIBC characterized by differential BCG responses, recurrence rates, and immune microenvironment composition (48). These subtypes correspond to distinct transcriptional states driven by chromatin remodeling and histone modification, implying that DNA methylation functions as both a biomarker and a driver of tumor evolution. Our subgroup findings, which highlight pathway-specific methylation effects on progression and survival, resonate strongly with these broader integrative models. The modest funnel plot asymmetry and significant Egger’s intercepts observed for PFS and RFS (p < 0.001) suggest the presence of small-study effects, potentially reflecting publication bias toward positive findings. However, sensitivity analyses demonstrated that exclusion of any single study did not materially alter the pooled estimates, confirming the robustness of the associations. Variation in detection platforms (MSP, qMSP, and pyrosequencing), cutoff thresholds, and clinical endpoints may explain residual heterogeneity. Future studies employing standardized methylation quantification, unified definitions of recurrence and progression, and multi-gene predictive panels will be critical for translating these findings into clinically actionable tools.

## Conclusion

This meta-analysis demonstrates that promoter DNA methylation emerges as a robust prognostic biomarker in non-muscle-invasive bladder cancer, with methylated cases showing a nearly threefold higher risk of recurrence and progression. Although overall survival effects were pathway-specific, stronger associations were observed in Asian cohorts, highlighting regional epigenetic variability. Standardization of validated gene panels, quantitative thresholds, and reproducible assays, combined with multi-ethnic prospective validation, will be critical for clinical translation. Integrating methylation-based classifiers into existing European Association of Urology and American Urological Association prognostic models may substantially improve individualized risk stratification and guide therapy selection in NMIBC.

## Data Availability

The original contributions presented in the study are included in the article/Supplementary Material; further inquiries can be directed to the corresponding author.

## References

[1] Siegel RL Miller KD Fuchs HE Jemal A . Cancer statistics, 2022. CA Cancer J Clin (2022) 72(1):7–33. 10.3322/caac.21708 35020204

[2] Cassell A Yunusa B Jalloh M Mbodji MM Diallo A Ndoye M Non-muscle invasive bladder cancer: a review of the current trend in Africa. World J Oncol (2019) 10(3):123–31. 10.14740/wjon1210 31312279 PMC6615913

[3] Singh MK Jain M Shyam H Shankar P Singh V . Associated pathogenesis of bladder cancer and SARS-CoV-2 infection: a treatment strategy. Virusdisease (2021) 32(4):613–5. 10.1007/s13337-021-00742-y 34604475 PMC8475482

[4] Singh V Singh MK Jain M Pandey AK Kumar A Sahu DK . The relationship between BCG immunotherapy and oxidative stress parameters in patients with nonmuscle invasive bladder cancer. Urol Oncol Semin Original Invest (2023) 41:486.e25–486.e32. 10.1016/j.urolonc.2023.09.008 37932135

[5] Jain M Mishra A Singh MK Shyam H Kumar S Shankar P Immunotherapeutic and their immunological aspects: current treatment strategies and agents. Natl J Maxill Surg (2022) 13(3):322–9. 10.4103/njms.njms_62_22 36683928 PMC9851344

[6] Claps F Pavan N Ongaro L Tierno D Grassi G Trombetta C BCG-unresponsive non-muscle-invasive bladder cancer: current treatment landscape and novel emerging molecular targets. Int J Mol Sci (2023) 24(16):12596. 10.3390/ijms241612596 37628785 PMC10454200

[7] Singh V Singh MK Kumar A Sahu DK Jain M Pandey AK Metabolomic biomarkers for prognosis in non-muscle invasive bladder cancer: a comprehensive systematic review and meta-analysis. Indian J Clin Biochem (2024) 2024. 10.1007/s12291-024-01187-y 40123630 PMC11928707

[8] Kumar S Singh V Singh MK Sankhwar SN . Management of metastatic renal cell carcinoma in a tertiary care hospital. Cureus (2023) 15(2):e35623. 10.7759/cureus.35623 37007390 PMC10063926

[9] Kumar A Singh MK Singh V Shrivastava A Sahu DK Bisht D The role of autophagy dysregulation in low and high-grade nonmuscle invasive bladder cancer: a survival analysis and clinicopathological association. Urol Oncol Semin Original Invest (2024) 42:452.e1–452.e13. 10.1016/j.urolonc.2024.07.017 39256148

[10] Kumar A Singh VK Singh V Singh MK Shrivastava A Sahu DK . Evaluation of fibroblast growth factor receptor 3 (FGFR3) and tumor protein P53 (TP53) as independent prognostic biomarkers in high-grade non-muscle invasive bladder cancer. Cureus (2024) 16(7):e65816. 10.7759/cureus.65816 39219882 PMC11362872

[11] Singh MK Singh V Kumar A . 101P survival analysis and clinicopathological correlation with molecular classification in BCG-Nonresponsive Ta-T1 bladder cancer. ESMO Open (2025) 10:104262. 10.1016/j.esmoop.2025.104262

[12] Singh MK Singh V Kumar A . 285P genetic variation of the inflammatory cytokine interleukin 17 in non-muscle invasive bladder cancer. Ann Oncol (2024) 35:S1515. 10.1016/j.annonc.2024.10.305

[13] Jang HS Shin WJ Lee JE Do JT . CpG and Non-CpG methylation in epigenetic gene regulation and brain function. Genes (Basel) (2017) 8(6):148. 10.3390/genes8060148 28545252 PMC5485512

[14] Bilgrami SM Qureshi SA Pervez S Abbas F . Promoter hypermethylation of tumor suppressor genes correlates with tumor grade and invasiveness in patients with urothelial bladder cancer. SpringerPlus (2014) 3:178. 10.1186/2193-1801-3-178 24790823 PMC4000596

[15] Singh V Madeshiya AK Ansari NG Singh MK Abhishek A . CYP1A1 gene polymorphism and heavy metal analyses in benign prostatic hyperplasia and prostate cancer: an explorative case-control study. Urol Oncol Semin Original Invest (2023) 41:355.e9–355.e17. 10.1016/j.urolonc.2023.04.022 37277283

[16] Hervouet E Cheray M Vallette FM Cartron PF . DNA methylation and apoptosis resistance in cancer cells. Cells (2013) 2(3):545–73. 10.3390/cells2030545 24709797 PMC3972670

[17] Poojary SS Singh MK . Chapter 3 - tumor cell metabolism and autophagy as therapeutic targets. In: Kumar D Asthana S , editors. Autophagy and metabolism. Academic Press (2022). p. 73–107.

[18] Oyaert M Van Praet C Delrue C Speeckaert MM . Novel urinary biomarkers for the detection of bladder cancer. Cancers (2025) 17(8):1283. 10.3390/cancers17081283 40282460 PMC12025552

[19] Haddaway NR Page MJ Pritchard CC McGuinness LA . PRISMA2020: an R package and shiny app for producing PRISMA 2020-compliant flow diagrams, with interactivity for optimised digital transparency and open synthesis. Campbell Syst Rev (2022) 18(2):e1230. 10.1002/cl2.1230 36911350 PMC8958186

[20] Alvarez-Mugica M Cebrian V Fernandez-Gomez JM Fresno F Escaf S Sanchez-Carbayo M . Myopodin methylation is associated with clinical outcome in patients with T1G3 bladder cancer. J Urol (2010) 184(4):1507–13. 10.1016/j.juro.2010.05.085 20723929

[21] Ha YS Kim JS Yoon HY Jeong P Kim TH Yun SJ Novel combination markers for predicting progression of nonmuscle invasive bladder cancer. Int J Cancer (2012) 131(4):E501–7. 10.1002/ijc.27319 22025348

[22] Jeong P Min BD Ha YS Song PH Kim IY Ryu KH RUNX3 methylation in normal surrounding urothelium of patients with non-muscle-invasive bladder cancer: potential role in the prediction of tumor progression. Eur J Surg Oncol (2012) 38(11):1095–100. 10.1016/j.ejso.2012.07.116 22884471

[23] Kim YJ Yoon HY Kim JS Kang HW Min BD Kim SK HOXA9, ISL1 and ALDH1A3 methylation patterns as prognostic markers for nonmuscle invasive bladder cancer: array-based DNA methylation and expression profiling. Int J Cancer (2013) 133(5):1135–42. 10.1002/ijc.28121 23436614

[24] Kim JS Chae Y Ha YS Kim IY Byun SS Yun SJ Ras association domain family 1A: a promising prognostic marker in recurrent nonmuscle invasive bladder cancer. Clin genitourinary Cancer (2012) 10(2):114–20. 10.1016/j.clgc.2011.12.003 22382007

[25] Kim YW Yoon HY Seo SP Lee SK Kang HW Kim WT Clinical implications and prognostic values of prostate cancer susceptibility candidate methylation in primary nonmuscle invasive bladder cancer. Dis Markers (2015) 2015:1–6. 10.1155/2015/402963 26074659 PMC4444592

[26] Kim YJ Kang HW Seo SP Jang H Kim T Kim WT RSPH9 methylation pattern as a prognostic indicator in patients with non-muscle invasive bladder cancer. J Urol (2016) 195(4):e1131. 10.3892/or.2015.4409 26575865

[27] Ma JG Xie PG Ma JG . Aberrant methylation of CDH13 is a potential biomarker for predicting the recurrence and progression of non-muscle-invasive bladder cancer. Med Sci Monitor (2014) 20, 1572–7. 10.12659/msm.892130 PMC4162450 25196672

[28] Lin YL Wang YL Ma JG Li WP . Clinical significance of protocadherin 8 (PCDH8) promoter methylation in non-muscle invasive bladder cancer. J Exp and Clin Cancer Res (2014) 33(1):68. 10.1186/preaccept-1759254111327153 25927589 PMC4237820

[29] van Kessel KEM van der Keur KA Dyrskjot L Algaba F Welvaart NYC Beukers W Molecular markers increase precision of the european association of urology non-muscle-invasive bladder cancer progression risk groups. Clin Cancer Res (2018) 24(7):1586–93. 10.1158/1078-0432.ccr-17-2719 29367430

[30] Sacristan R Gonzalez C Fernández-Gómez JM Fresno F Escaf S Sánchez-Carbayo M . Molecular classification of Non–muscle-invasive bladder cancer (PTa low-grade, pT1 low-grade, and pT1 high-grade subgroups) using methylation of tumor-suppressor genes. J Mol Diagn (2014) 16(5):564–72. 10.1016/j.jmoldx.2014.04.007 24998186

[31] Stang A . Critical evaluation of the Newcastle-Ottawa scale for the assessment of the quality of nonrandomized studies in meta-analyses. Eur J Epidemiol (2010) 25(9):603–5. 10.1007/s10654-010-9491-z 20652370

[32] Lee SH Hyeon DY Yoon SH Jeong JH Han SM Jang JW RUNX3 methylation drives hypoxia-induced cell proliferation and antiapoptosis in early tumorigenesis. Cell Death and Differ (2021) 28(4):1251–69. 10.1038/s41418-020-00647-1 33116296 PMC8027031

[33] Rubina K Maier A Klimovich P Sysoeva V Romashin D Semina E T-Cadherin (CDH13) and non-coding RNAs: the crosstalk between health and disease. Int J Mol Sci (2025) 26(13):6127. 10.3390/ijms26136127 40649905 PMC12250294

[34] Katto J Mahlknecht U . Epigenetic regulation of cellular adhesion in cancer. Carcinogenesis (2011) 32(10):1414–8. 10.1093/carcin/bgr120 21705481

[35] Yu H Jiang X Jiang L Zhou H Bao J Zhu X Protocadherin 8 (PCDH8) inhibits proliferation, migration, invasion, and angiogenesis in esophageal squamous cell carcinoma. Med Sci Monitor (2020) 26:e920665. 10.12659/msm.920665 32330123 PMC7197227

[36] Noeraparast M Krajina K Pichler R Niedersüß‐Beke D Shariat SF Grunwald V FGFR3 alterations in bladder cancer: sensitivity and resistance to targeted therapies. Cancer Commun (London, England) (2024) 44(10):1189–208. 10.1002/cac2.12602 39161208 PMC11483561

[37] Chiba K Lorbeer FK Shain AH McSwiggen DT Schruf E Oh A Mutations in the promoter of the telomerase gene TERT contribute to tumorigenesis by a two-step mechanism. Science (2017) 357(6358):1416–20. 10.1126/science.aao0535 28818973 PMC5942222

[38] Heidenreich B Kumar R . TERT promoter mutations in telomere biology. Mutat Res Rev Mutat Res (2017) 771:15–31. 10.1016/j.mrrev.2016.11.002 28342451

[39] Gabrielson A Wu Y Wang H Jiang J Kallakury B Gatalica Z Intratumoral CD3 and CD8 T-cell densities associated with relapse-free survival in HCC. Cancer Immunol Res (2016) 4(5):419–30. 10.1158/2326-6066.cir-15-0110 26968206 PMC5303359

[40] Wang B Wu S Zeng H Liu Z Dong W He W CD103+ tumor infiltrating lymphocytes predict a favorable prognosis in urothelial cell carcinoma of the bladder. J Urol (2015) 194(2):556–62. 10.1016/j.juro.2015.02.2941 25752441

[41] Hewavisenti R Ferguson A Wang K Jones D Gebhardt T Edwards J CD103+ tumor-resident CD8+ T cell numbers underlie improved patient survival in oropharyngeal squamous cell carcinoma. J Immunother Cancer (2020) 8(1):e000452. 10.1136/jitc-2019-000452 32527931 PMC7292045

[42] Ke HL Lin J Ye Y Wu WJ Lin HH Wei H Genetic variations in glutathione pathway genes predict cancer recurrence in patients treated with transurethral resection and bacillus calmette-guerin instillation for non-muscle invasive bladder cancer. Ann Surg Oncol (2015) 22(12):4104–10. 10.1245/s10434-015-4431-5 25851338 PMC4598273

[43] Witjes JA Morote J Cornel EB Gakis G van Valenberg FJP Lozano F Performance of the bladder EpiCheck methylation test for patients under surveillance for non-muscle-invasive bladder cancer: results of a multicenter, prospective, blinded clinical trial. Eur Urol Oncol (2018) 1(4):307–13. 10.1016/j.euo.2018.06.011 31100252

[44] Ferro M La Civita E Liotti A Cennamo M Tortora F Buonerba C Liquid biopsy biomarkers in urine: a route towards molecular diagnosis and personalized medicine of bladder cancer. J personalized Med (2021) 11(3):237. 10.3390/jpm11030237 33806972 PMC8004687

[45] Beijert IJ van den Burgt Y Hentschel AE Bosschieter J Kauer PC Lissenberg-Witte BI Bladder cancer detection by urinary methylation markers GHSR/MAL: a validation study. World J Urol (2024) 42(1):578. 10.1007/s00345-024-05287-5 39412544 PMC11485176

[46] Kim E Zucconi BE Wu M Nocco SE Meyers DJ McGee JS MITF expression predicts therapeutic vulnerability to p300 inhibition in human melanoma. Cancer Res (2019) 79(10):2649–61. 10.1158/0008-5472.can-18-2331 30910803 PMC6522293

[47] Weeber F Ooft SN Dijkstra KK Voest EE . Tumor organoids as a pre-clinical cancer model for drug discovery. Cell Chem Biol (2017) 24(9):1092–100. 10.1016/j.chembiol.2017.06.012 28757181

[48] Falick Michaeli T Azria B Lotem M Friedman N . Elucidating the epigenetic landscape of non-muscle invasive bladder cancer. J Clin Oncol (2025) 43(Suppl. l):e16580. 10.1200/jco.2025.43.16_suppl.e16580

