# Supplementary material for: DNA methylation as prognostic factors in non-muscle-invasive bladder cancer: a systematic review and meta-analysis
Source: Oncol Rev. 2025 Nov 25;19:1679974. doi: 10.3389/or.2025.1679974 (PMC12685936; doi:10.3389/or.2025.1679974)
Supplement: Supplementary file 1 [file Supplementaryfile1.docx]

| **Study Excluded** | **Pooled HR (95% CI)** | **Heterogeneity (I²%)** | **p-value** |
| --- | --- | --- | --- |
| **All studies** | 2.88 (2.03-4.09) | 36 | 0.09 |
| **Ha 2012 (RUNX3)** | 2.75 (1.96-3.87) | 33 | 0.12 |
| **Jeong 2012 (RUNX3)** | 2.82 (1.96-4.04) | 38 | 0.08 |
| **Kim JS 2012 (RASSF1A)** | 2.74 (1.94-3.89) | 35 | 0.11 |
| **Lin 2014 (CDH13)** | 2.64 (1.87-3.73) | 30 | 0.14 |
| **Alvarez-Mugica 2010 (Myopodin)** | 3.00 (2.03-4.46) | 41 | 0.06 |
| **Kim YW 2015 (PRAC)** | 2.78 (1.96-3.95) | 36 | 0.09 |
| **Lin 2014 (PCDH8)** | 2.74 (1.90-3.95) | 35 | 0.1 |
| **Kim YJ 2013 (ALDH1A3, ISL1)** | 2.90 (1.94-4.34) | 44 | 0.05 |
| **Kim YJ 2016 (RSPH9)** | 2.78 (1.95-3.95) | 36 | 0.09 |
| **Van Kessel 2018 (GATA2, TBX2/3, ZIC4)** | 4.37 (2.97-6.45) | 0 | 0.75 |

Table S1: Sensitivity for the methylation effect in PFS

| **Study Excluded** | **Pooled HR (95% CI)** | **Heterogeneity (I²%)** | **p-value** |
| --- | --- | --- | --- |
| **All studies** | 2.65 (1.93-3.63) | 49 | 0.04 |
| **Ha 2012 (RUNX3)** | 2.43 (1.87-3.16) | 32 | 0.16 |
| **Kim JS 2012 (RASSF1A)** | 2.58 (1.88-3.55) | 51 | 0.04 |
| **Lin 2014 (CDH13)** | 2.46 (1.80-3.35) | 45 | 0.07 |
| **Sacristan 2014 (PAX5a, RB1)** | 3.10 (2.11-4.55) | 42 | 0.1 |
| **Alvarez-Mugica 2010 (Myopodin)** | 2.53 (1.87-3.43) | 47 | 0.06 |
| **Kim YW 2015 (PRAC)** | 2.69 (1.89-3.83) | 54 | 0.03 |
| **Lin 2014 (PCDH8)** | 2.78 (1.89-4.08) | 54 | 0.03 |
| **Kim YJ 2013 (HOXA9)** | 2.88 (2.00-4.15) | 52 | 0.03 |
| **Kim YJ 2016 (RSPH9)** | 2.64 (1.85-3.76) | 52 | 0.03 |

Table S2: Sensitivity for the methylation effect in RFS


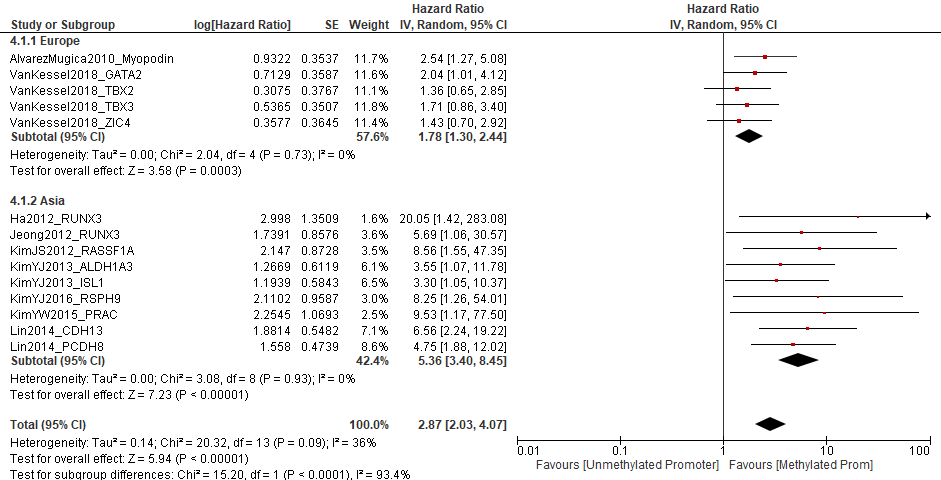


Figure S1. Forest plot of subgroup analysis for progression-free survival according to study geography


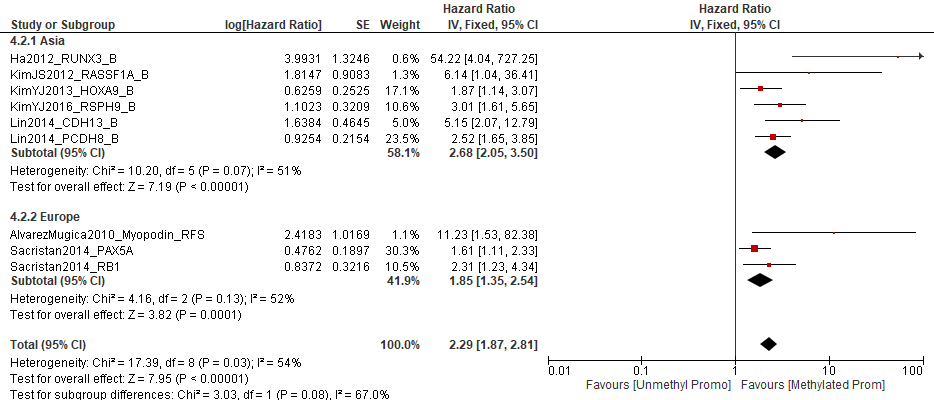


Figure S2. Forest plot showing subgroup analysis for RFS by geographical region


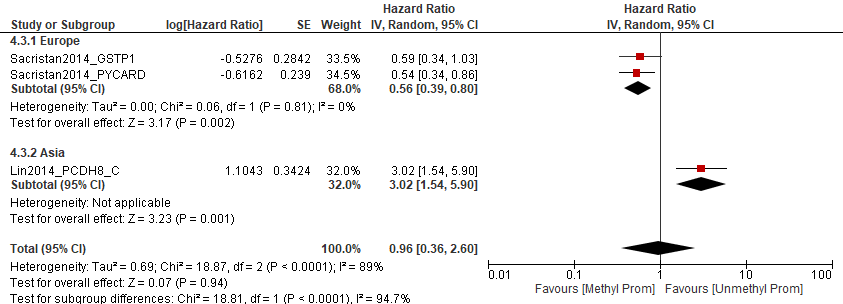


Figure S3. Forest plot showing regional subgroup analysis for overall survival in NMIBC


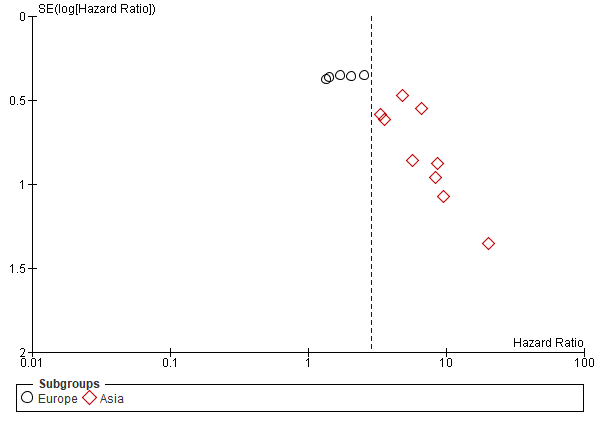


**Figure S4.** Funnel plot showing regional publication bias for progression-free survival (PFS) in NMIBC


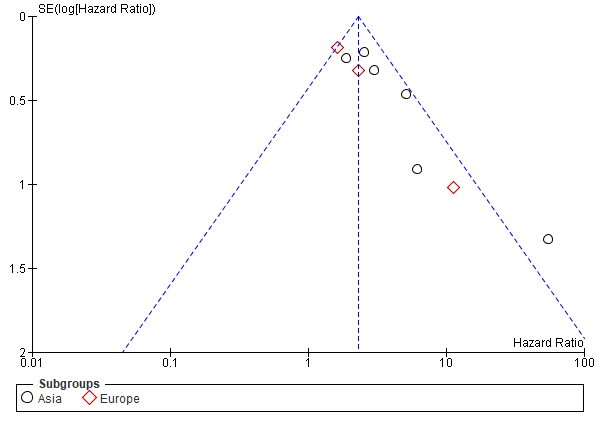


**Figure S5.** Funnel plot showing regional publication bias for recurrence-free survival (RFS) in NMIBC


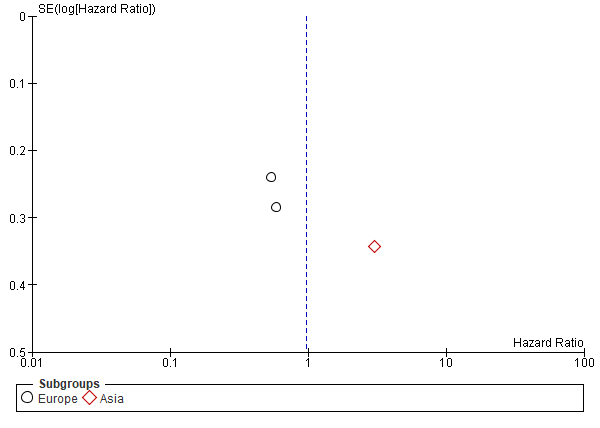


**Figure S6.** Funnel plot showing regional publication bias for overall survival (OS) in NMIBC
